# Current News

**Published:** 1903-08

**Authors:** 


### Current News.

### AMERICAN SOCIETY OF ORTHODONTISTS.

THE third annual meeting of the American Society of Orthodontists will be held on December 30 and 31, 1903, and January 1, 1904, in Buffalo, N. Y.

ANNA HOPKINS,
*Secretary.*

ST. LOUIS, MO.

---

### MISSOURI STATE DENTAL ASSOCIATION.

AT the thirty-ninth annual meeting of the Missouri State Dental Association, held at Kansas City, May 19 to 21, 1903, the following officers were elected:

President, J. H. Kennerly, St. Louis; First Vice-President, F. W. Franklin, Kansas City; Second Vice-President, F. H. Achelpohl, St. Charles; Recording Secretary, H. H. Sullivan, Kansas

City; Corresponding Secretary, S. T. Bassett, St. Louis; Treasurer, J. F. Fry, Moberly.

*Board of Censors.*—J. C. Pasqueth, Mexico; A. L. Bridgeford, Macon; De Courcey Lindsley, St. Louis.

*Committee on Ethics.*—J. B. McBride, Springfield; J. A. Prosser, St. Louis.

*Committee on Publication.*—W. G. Goodrich, Chillicothe; Otto J. Fruth, St. Louis.

*Committee on New Appliances.*—J. F. Austin, St. Louis.

The next meeting will be held at St. Louis, third Tuesday in May, 1904.

SAM T. BASSETT,
*Corresponding Secretary.*

---

### HARVARD DENTAL ALUMNI ASSOCIATION.

AT the thirty-second annual meeting of the Harvard Dental Alumni Association, held in Boston, Mass., Monday, June 22, 1903, the following officers were elected for the ensuing year:

President, Charles E. Perkins, '90, Brockton, Mass.; Vice-President, Arthur W. Stoddard, '87, Boston, Mass.; Secretary, Waldo E. Boardman, '86, Boston, Mass.; Treasurer, E. Proctor Holmes, '88, Boston, Mass.

*Executive Committee.*—Waldo E. Boardman, '86, *ex-officio* Chairman, Boston Mass.; William P. Cooke, '81, for two years, Boston, Mass.; Harry S. Parsons, '92, for one year, Boston, Mass.

WALDO E. BOARDMAN, '86,
*Secretary.*

---

### MINNESOTA STATE DENTAL ASSOCIATION.

THE twentieth annual meeting of the Minnesota State Dental Association will be held at the Dental Department of the State University in Minneapolis, on Tuesday, Wednesday, and Thursday, September 1, 2, and 3, 1903. All dentists are cordially invited to attend.

GEO. S. TODD,
*Secretary.*

LAKE CITY, MINN.

